# Recognizing muscle health in healthy aging and non-communicable disease policy: a public health perspective

**DOI:** 10.3389/fpubh.2026.1913349

**Published:** 2026-08-27

**Authors:** Alvaro Puelles-Diaz

**Affiliations:** 1 Department of Kinesiology, Faculty of Sciences, University of La Serena, La Serena, Chile; 2 School of Medicine, Universidad del Alba, La Serena, Chile

**Keywords:** health policy, healthy aging, muscle health, muscle strength, non-communicable diseases, resistance training, sarcopenia

## Abstract

Population aging and the rising burden of non-communicable diseases (NCDs) place sustained pressure on health systems. In this perspective, I argue that muscle health—muscle mass, strength, and physical function—should be recognized as an explicit and prognostically distinct target within policies for healthy aging and NCD prevention. Two arguments distinguish this from generic physical activity promotion: muscle strength predicts mortality, regardless of activity level, and the most significant gains are produced by resistance and multicomponent training rather than by aerobic activity alone. I summarize randomized evidence, including trials with follow-up to 36 months, highlighting that the benefits are more pronounced among individuals with demonstrable functional impairment—a finding that strengthens, rather than weakens, the policy case. I identify three recurring policy gaps: the limited use of feasible assessment tools in primary care, fragmented prevention efforts that under-prioritize resistance training, and the near absence of functional capacity in global NCD frameworks. Drawing on experiences from Japan, Europe, North America, and Brazil, I propose five operational priorities and examine the practical constraints on delivery—equipment, financing, workforce, cultural barriers, and long-term adherence—alongside the limits of the economic evidence, so that policy ambition is matched to the strength of the data.

## 1 Introduction

Population aging and the rising prevalence of non-communicable diseases (NCDs) are among the most pressing challenges for health systems, with NCDs responsible for the majority of deaths worldwide and a burden that rises sharply with age (1, 2). Skeletal muscle, long regarded as a contractile tissue for locomotion and energy metabolism, is now also understood as an endocrine organ that secretes myokines influencing systemic physiology (3, 4).

It helps to be precise about terms. Recent consensus work—the Global Leadership Initiative in Sarcopenia (GLIS) and the revised European Working Group on Sarcopenia in Older People criteria—frames the condition around the combination of low muscle mass and low muscle strength or physical function (5, 6). I use *muscle health* in this sense: mass, strength, and function (for example, grip strength and gait speed). This construct overlaps with physical activity but is not the same thing.

That distinction is the crux of the policy argument, so it is worth defending directly. First, muscle health carries prognostic information that activity levels do not capture: in the multinational Prospective Urban Rural Epidemiology (PURE) cohort, grip strength predicted all-cause and cardiovascular mortality after adjustment for physical activity and other risk factors, and was a stronger predictor of death than systolic blood pressure (7). Second, the interventions differ: the gains in strength and muscle mass associated with better outcomes are produced reliably by resistance and multicomponent training, not by aerobic activity promotion alone. Treating muscle health as an explicit target is therefore not a relabeling of existing physical activity policy but a case for measuring a prognostically distinct determinant and for prioritizing the specific interventions that improve it.

The downstream consequences of poor muscle health are well documented for several established outcomes. Low muscle mass and strength are associated with frailty, falls, physical disability, loss of independence, type 2 diabetes, and higher rates of hospitalization and adverse postoperative outcomes (6, 8). A muscle–brain axis has also drawn attention, and some cohorts report associations between higher muscle mass and lower risk of neurodegenerative decline, including Alzheimer’s disease (9–11); this neurocognitive evidence is more tentative and largely observational, and I treat it as a hypothesis-generating signal rather than a basis for policy.

**Figure 1:**
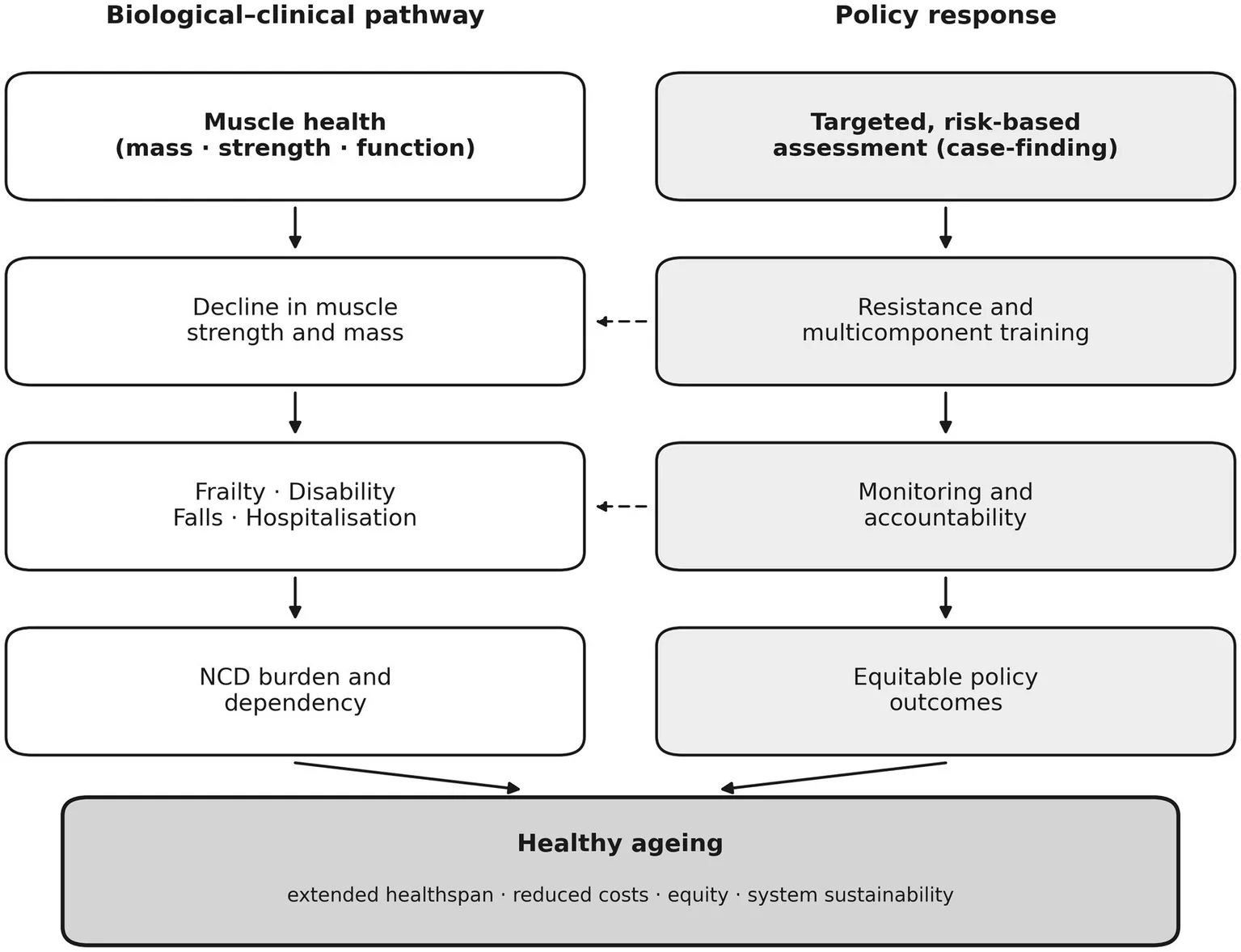
Muscle health as a policy lever for healthy aging. The biological–clinical pathway (left) links muscle health—mass, strength, and function—to its decline and to frailty, disability, falls, hospitalization, and ultimately non-communicable disease (NCD) burden and dependency. The policy response (right)—risk-based assessment, resistance and multicomponent training, and monitoring—acts on that pathway (dashed arrows) so that both converge on healthy aging.

Accordingly, this article advances a viewpoint: that muscle health should be embedded explicitly in research agendas and policy frameworks for aging and NCDs. As a Perspective, it is a viewpoint rather than a systematic review; the evidence cited was selected to illustrate biological plausibility, prognostic relevance, trial findings, and policy and implementation experience, and to mark where the evidence base remains thin. Figure 1 summarizes the argument, linking muscle health to healthy aging outcomes and the policy actions that act on that pathway.

## 2 Biological rationale in brief

Through its secretome, skeletal muscle releases myokines—including interleukin-6 (IL-6), insulin-like growth factor-1 (IGF-1), and myostatin—that participate in glucose uptake, lipid metabolism, protein synthesis, and interorgan communication (3, 4).

Exercise is associated with favorable epigenetic modifications, such as DNA hypomethylation and histone acetylation, that accompany improved mitochondrial biogenesis, oxidative capacity, and insulin sensitivity (12, 13). These adaptations plausibly contribute to cardiometabolic resilience and underscore the preventive potential of muscle-preserving interventions across the lifespan.

Conversely, muscle atrophy—from aging, inactivity, or disease—accompanies insulin resistance and chronic low-grade inflammation, and tracks with frailty, disability, and poorer outcomes in diabetes, cardiovascular disease, and cancer (6, 8, 14). Collectively, this evidence positions skeletal muscle as both a candidate biomarker and a modifiable target for public health strategy, while acknowledging that much of the mechanistic human evidence is associative (Table 1).

**Table 1:** An operational policy framework for muscle health.

Pillar	Action	Lead and delivery	Minimum indicator
1. Assessment	Case-finding in at-risk groups using handgrip and gait speed (BIA/ultrasound where standardized), with validated cut-points	Health ministry/primary care services; nurses and physicians, with kinesiology/physiotherapy support	% of at-risk adults assessed; detection rate
2. Community prevention	Expand progressive resistance and multicomponent programs in municipal, workplace, and community settings	Municipalities; community instructors and exercise professionals	Coverage; adherence at 6 and 12 months; change in strength/function
3. Research and implementation	Fund pragmatic trials with long-term follow-up and economic evaluation	Research funders; academic and health-service partnerships	Trials funded; cost-effectiveness estimates produced
4. Equity and adaptation	Subsidize participation in underserved areas; adapt to rural/remote contexts and local norms	Social-protection and health authorities; civil society	Indicators disaggregated by sex, age, geography, income
5. Monitoring and governance	Align with GAPPA indicators; publish dashboards; tie funding to targets	Health ministry/national statistics office	Public reporting; budget linked to targets

BIA, bioimpedance analysis; GAPPA, Global Action Plan on Physical Activity. Leads and delivery models are illustrative and should be adapted to national governance and financing structures.

## 3 What the randomized evidence shows: effects, heterogeneity, and limits

Randomized trials support the claim that muscle-targeted interventions change function, and they also define the boundaries of that claim.

*Effects*. The SPRINTT trial randomized 1,519 community-dwelling adults aged 70 years or older with physical frailty and sarcopenia, across 16 sites in 11 European countries, to a multicomponent intervention—supervised moderate-intensity physical activity twice weekly at a center, home-based sessions, and nutritional counseling—or to healthy-aging education, with follow-up to 36 months; the intervention reduced incident mobility disability among participants whose baseline short physical performance battery (SPPB) score was 3 to 7 (15). The LIFE trial randomized 1,635 sedentary adults aged 70 to 89 years with functional limitation to structured walking, resistance, and flexibility training or to health education, and over a mean of 2.6 years reduced major mobility disability (30.1% versus 35.5%; hazard ratio 0.82, 95% confidence interval 0.69 to 0.98) (16). Multicenter randomized evaluation of the Vivifrail multicomponent program likewise reports improved functional capacity in frail older adults (17).

*Heterogeneity of interventions*. Programs grouped under “resistance” or “multicomponent” training differ in modality, intensity, frequency, duration, supervision, and nutritional co-intervention, and those differences change results. A network meta-analysis of 42 randomized trials in 3728 adults with sarcopenia found resistance exercise—alone or combined with balance, aerobic training, or nutrition—to be the most effective approach overall, but the best-performing combination varied by outcome: resistance with balance training and nutrition ranked highest for handgrip strength, while resistance-based combinations ranked highest for gait speed and quality of life (18). Policy should therefore name a modality—progressive resistance training, with balance and nutritional support where feasible—rather than endorse exercise generically.

*Long-term effects and apparently conflicting findings*. Two caveats matter. First, durability is understudied: median follow-up across sarcopenia exercise trials is approximately 12 weeks (18), which makes the multi-year LIFE and SPRINTT results the exception rather than the norm. Second, benefit is not uniform. In SPRINTT, no reduction in mobility disability was observed among participants with better baseline function (SPPB 8 or 9; hazard ratio 1.25, 95% confidence interval 0.79 to 1.95) (15); in LIFE, the intervention arm showed a non-significant excess of hospitalizations16; and pooled analyses find that adherence, although high in trials, is not straightforwardly associated with efficacy (19). I read these results as specifying the policy case rather than undermining it. The evidence is strongest where functional impairment is already demonstrable, and weakest for undifferentiated, population-wide prescription. That conclusion argues for risk-based case-finding, set out below, and against universal screening.

## 4 Policy challenges and missed opportunities

Three recurring gaps limit translation: (i) a measurement gap in primary care, where feasible tools are rarely embedded in workflows; (ii) fragmented prevention, with underinvestment in structured, muscle-centric programs despite the World Health Organization (WHO) Global Action Plan on Physical Activity (GAPPA) (20); and (iii) limited integration of muscle health into NCD “Best Buys,” where tobacco, alcohol, and diet dominate while functional capacity remains neglected (1).

- *Measurement gap*. Validated, low-cost instruments exist, but their use is not standardized, contributing to late detection of sarcopenia and functional decline (6, 21). Pragmatic pilots—such as embedding exercise scientists in primary care—suggest that routine measurement can improve detection and adherence (22).
- *Fragmented prevention*. Physical-activity promotion is widespread, but structured, muscle-preserving approaches remain under-prioritized, despite randomized evidence of benefit for function and disability (15, 16, 18). Community-scale initiatives such as Brazil’s Academia da Saúde show that targeted investment can yield measurable dividends (23).
- *Limited integration*. Global action plans emphasize tobacco, alcohol, and diet, but functional capacity—despite its predictive value for disability and cost—remains largely absent from core strategies (1). Comparative analyses argue that musculoskeletal and muscle health deserve greater priority in aging and NCD frameworks (24). Together, these gaps reflect a systemic underrecognition of skeletal muscle as a strategic health asset.

## 5 International experiences and lessons

*Japan*. Health Japan 21 operationalizes activity and nutrition targets with measurable indicators; in its evaluations, gains in healthy life expectancy outpaced gains in total life expectancy (25).

*Europe*. WHO healthy-aging frameworks place functional ability at the center of policy (26). Denmark and the Netherlands have introduced community-based resistance-training programs for older adults, supported by municipal funding and primary-care referral (21, 27).

*North America*. Canada’s physical-activity guidelines for older adults include resistance and balance training, and the United States’ Medicare-supported SilverSneakers benefit illustrates how financial incentives can increase structured activity among older adults (28).

*Low- and middle-income countries (LMICs)*. Implementation is uneven, constrained by financing and monitoring (24). Brazil’s Academia da Saúde shows that low-cost infrastructure and community instructors can expand access to resistance training within universal health coverage (23).

*Lesson*. Global frameworks exist, but success depends on adaptation to local context, financing, and workforce structures.

## 6 Toward a muscle-health policy framework

Treating skeletal muscle as a population health asset with positive externalities—a determinant of healthy aging whose benefits extend beyond the individual—suggests five priorities.

- *Targeted assessment rather than blanket screening*. Handgrip strength and gait speed are the most feasible first-line measures in primary care (22, 26); bioimpedance and, especially, muscle ultrasound add information but are not yet internationally standardized, with protocols and cut-points still varying between settings (5, 21). The case is strongest for case finding among higher-risk groups—older adults, people with chronic disease or recent functional change—rather than universal population screening, and these are precisely the groups in which randomized benefit has been demonstrated (15, 16). Three conditions warrant attention before any routine program: validated, context-appropriate cut-points (5, 6); the risk of overdiagnosis and medicalization of normal aging; and the primary-care workforce and time required.
- *Scaling community programs*. Progressive resistance and multicomponent training improve function and reduce mobility disability (15, 16, 18); Brazil and Denmark offer scalable, low-cost models (23, 27).
- *Translational pipelines*. Myokine and epigenetic research (3, 12) should inform pragmatic trials and implementation toolkits linking muscle biology to clinically and economically meaningful outcomes (29), with follow-up long enough to establish durability.
- *Embedding in existing frameworks*. Functional capacity is largely missing from NCD Best Buys (1) and under-weighted in aging strategies (26); explicit inclusion would make muscle health a measurable target without new vertical programs.
- *Monitoring and accountability*. Japan’s measurable targets show the value of indicators tied to evaluation (25). Standardized muscle and function indicators in surveys and records would enable return on investment analysis over time.

*A realistic appraisal of returns*. Muscle health is plausibly a high-value lever. Low muscle strength, sarcopenia, and frailty are associated with higher hospitalization and care costs: in a recent European cohort, frailty was associated with approximately 24% higher annual hospitalization costs than non-frailty, while among people with sarcopenia, concomitant frailty was associated with 46–56% higher annual hospitalization costs than sarcopenia without frailty, depending on the frailty definition used (30). An earlier US analysis attributed about US$18.5 billion in direct healthcare costs—around 1.5% of health expenditure—to sarcopenia, with an estimated US$1 billion in annual savings from a 10% reduction in prevalence (31). A systematic review found higher costs among sarcopenic patients in 11 of 14 studies, while cautioning that the evidence is heterogeneous and methodologically limited (32); the cost of physical inactivity to health systems is also well documented (20). The honest reading is that the economic case is promising but not settled: direct cost-effectiveness data for muscle-specific assessment and programs remain sparse, and large fiscal claims should be framed as hypotheses for trials and economic evaluation rather than as established savings.

## 7 Implementation challenges

Recommending assessment and training is easier than delivering them. Five constraints recur, and each has a policy response.

*Equipment availability*. Handgrip dynamometry is inexpensive and portable, but requires calibration, maintenance, and trained use. Bioimpedance and muscle ultrasound need capital investment and servicing, and ultrasound additionally requires operator training; neither has yet internationally agreed protocols or cut-points (5, 21). Where equipment cannot be sustained, gait speed and chair-stand tests need only a stopwatch and a measured corridor, and should be the default rather than a fallback.

*Financing*. Cost data are scarce but not absent: in LIFE, the structured program cost approximately US$1,270 per participant per year (US$3,302 over a mean follow-up of 2.6 years), based on direct intervention costs reported in the LIFE cost-effectiveness analysis (36). At the population scale, this is material. This argues for delivery through existing municipal and primary-care infrastructure rather than new vertical services, and for concentrating resources on the groups in which benefit has been demonstrated.

*Workforce and delivery*. Primary-care time is the binding constraint on assessment. Task-sharing helps: embedding exercise professionals in primary care improved detection and adherence in a cluster-randomized trial (22), and Brazil’s community-instructor model shows that delivery need not depend on scarce clinical staff (23).

*Cultural barriers*. Resistance training is unfamiliar or unappealing to many older adults. Systematic review evidence identifies beliefs that weight training is unsafe or inappropriate in later life, fear of injury, gendered perceptions of gym environments, and a preference for walking, alongside facilitators including supervision, social contact, and visible functional gain (33). Public messaging and program design must address these explicitly rather than assume uptake, and should be adapted to local norms about exercise in later life.

*Long-term adherence*. Adherence within trials is high—a meta-analysis of 17 randomized trials in 2975 adults with sarcopenia reported pooled adherence of 85% (95% confidence interval, 79–89%)—but it was lower in programs lasting 24 weeks or more, and most trials incorporated only a small number of behavior change techniques (19). Trial adherence also flatters what unsupervised community programs achieve. Because gains regress when training stops, policy should fund continuing provision rather than time-limited pilots, and should build in behavior change support, tapered supervision, and social components from the outset.

## 8 Adapting to local realities and advancing equity

Policy transfer must be context-sensitive. High-income settings may layer biomarker-informed personalization [for example, inflammatory or mitochondrial markers (14)] onto core programs, whereas resource-constrained settings should prioritize low-cost assessment and community delivery (23). WHO’s Decade of Healthy Aging emphasizes equity and multisectoral coalitions, and GAPPA calls for locally adaptable strategies mobilizing education, urban planning, and civil society (20, 34).

Equity deserves explicit design rather than assertion. Programs that rely on self-referral, private facilities, or digital tools can widen gaps for rural, lower-income, or low-literacy populations; conversely, community-instructor models integrated with universal coverage, such as Academia da Saúde, can narrow them (23). Three safeguards help: site programs where need is greatest rather than where uptake is easiest; subsidize participation and transport in underserved areas; and disaggregate monitoring indicators by sex, age, geography, and socioeconomic status, so that equity is measured rather than assumed.

## 9 Conclusion

Muscle health is a modifiable, prognostically distinct determinant of healthy aging with meaningful spillover benefits for disability prevention and NCD burden. Randomized evidence shows that multicomponent and resistance-based programs reduce mobility disability in older adults with demonstrable functional impairment, while also marking where the evidence is limited: durability beyond a few months, cost-effectiveness, and delivery at scale. By leveraging GAPPA (20), the Global Status Report on Physical Activity 2022 (35), the WHO Best Buys (1), and the Decade of Healthy Aging34, countries can institutionalize risk-based muscle assessment, scale resistance-based programs, and fund translational pipelines—while concurrently building the cost-effectiveness, adherence, and long-term outcome evidence still required. The payoff is healthier aging through policies that are effective, equitable, and locally adaptable.

## References

[1] World Health Organization. Tackling NCDs: 'best Buys' and other Recommended Interventions for the Prevention and Control of Noncommunicable Diseases. Geneva: WHO (2017).

[2] Beaglehole R Bonita R Horton R Adams C Alleyne G Asaria P . Priority actions for the non-communicable disease crisis. Lancet. (2011) 377:1438–47. doi: 10.1016/S0140-6736(11)60393-0, 21474174

[3] Pedersen BK Febbraio MA. Muscle as an endocrine organ: focus on muscle-derived interleukin-6. Physiol Rev. (2008) 88:1379–406. doi: 10.1152/physrev.90100.2007, 18923185

[4] Florin A Lambert C Sanchez C Zappia J Durieux N Tieppo AM . The secretome of skeletal muscle cells: a systematic review. Osteoarthr Cartil Open. (2020) 2:100019. doi: 10.1016/j.ocarto.2019.100019, 36474563 PMC9718214

[5] Kirk B Cawthon PM Arai H Ávila-Funes JA Barazzoni R Bhasin S . The conceptual definition of sarcopenia: Delphi consensus from the global leadership initiative in sarcopenia (GLIS). Age Ageing. (2024) 53:afae052. doi: 10.1093/ageing/afae052, 38520141 PMC10960072

[6] Cruz-Jentoft AJ Sayer AA. Sarcopenia. Lancet. (2019) 393:2636–46. doi: 10.1016/S0140-6736(19)31138-9 31171417

[7] Leong DP Teo KK Rangarajan S Lopez-Jaramillo P Avezum A Orlandini A . Prognostic value of grip strength: findings from the prospective urban rural epidemiology (PURE) study. Lancet. (2015) 386:266–73. doi: 10.1016/S0140-6736(14)62000-6, 25982160

[8] Jun L Robinson M Geetha T Broderick TL Babu JR. Prevalence and mechanisms of skeletal muscle atrophy in metabolic conditions. Int J Mol Sci. (2023) 24:2973. doi: 10.3390/ijms24032973, 36769296 PMC9917738

[9] Burtscher J Millet GP Burtscher M. The muscle–brain axis in neurodegenerative diseases: the key role of physical activity and exercise. Mov Disord. (2021) 36:59–63. doi: 10.1002/mds.28310, 33026697 PMC7891378

[10] Duranti C Belli R Gori AM Galli A Baroni S Forti P . Muscle health in neurodegenerative diseases: from mechanisms to interventions. Int J Mol Sci. (2023) 24:10784. doi: 10.3390/ijms241310784 37445969 PMC10341785

[11] Kim G Cheon SY Cho HJ Yoon HJ Park H Kim HJ . Muscle mass and risk of Alzheimer's disease: a population-based cohort study. Sci Rep. (2019) 9:12323. doi: 10.1038/s41598-018-37244-931444408

[12] Plaza-Díaz J Izquierdo D Torres-Martos Á Baig AT Aguilera CM Ruiz-Ojeda FJ. Impact of physical activity and exercise on the epigenome in skeletal muscle and effects on systemic metabolism. Biomedicine. (2022) 10:126. doi: 10.3390/biomedicines10010126, 35052805 PMC8773693

[13] Ling C Rönn T. Epigenetics in human obesity and type 2 diabetes. Cell Metab. (2019) 29:1028–44. doi: 10.1016/j.cmet.2019.03.009, 30982733 PMC6509280

[14] Dalle S Rossmeislová L Koppo K. The role of inflammation in age-related sarcopenia. Front Physiol. (2017) 8:1045. doi: 10.3389/fphys.2017.01045, 29311975 PMC5733049

[15] Bernabei R Landi F Calvani R Cesari M Del Signore S Anker SD . Multicomponent intervention to prevent mobility disability in frail older adults: randomised controlled trial (SPRINTT project). BMJ. (2022) 377:e068788. doi: 10.1136/bmj-2021-068788, 35545258 PMC9092831

[16] Pahor M Guralnik JM Ambrosius WT Blair S Bonds DE Church TS . Effect of structured physical activity on prevention of major mobility disability in older adults: the LIFE study randomized clinical trial. JAMA. (2014) 311:2387–96. doi: 10.1001/jama.2014.5616, 24866862 PMC4266388

[17] Casas-Herrero Á Sáez de Asteasu ML Antón-Rodrigo I Sánchez-Sánchez JL Montero-Odasso M Marín-Epelde I . Effects of Vivifrail multicomponent intervention on functional capacity: a multicentre, randomized controlled trial. J Cachexia Sarcopenia Muscle. (2022) 13:884–93. doi: 10.1002/jcsm.12925 35150086 PMC8977963

[18] Shen Y Shi Q Nong K Li S Yue J Huang J . Exercise for sarcopenia in older people: a systematic review and network meta-analysis. J Cachexia Sarcopenia Muscle. (2023) 14:1199–211. doi: 10.1002/jcsm.13225, 37057640 PMC10235889

[19] Wu S Nan J Chang J Jiang D Cao Z Zhou S . Adherence to exercise intervention for community-dwelling older adults with sarcopenia: a systematic review and meta-analysis. Age Ageing. (2025) 54:afaf094. doi: 10.1093/ageing/afaf094 40253683

[20] World Health Organization. Global action plan on Physical Activity 2018–2030: more Active People for a Healthier World. Geneva: WHO (2018).

[21] Maier AB van der Plas S van Schooten KS van Lummel RC Meskers CGM. Sarcopenia in community-dwelling older adults: epidemiology, diagnosis, and clinical management. Eur Geriatr Med. (2023) 14:45–54. doi: 10.1007/s41999-022-00764-5

[22] Wattanapisit A Thanamee S Srichan W Petchuay P Putthinoi S Anusiri W . Effectiveness of the physical activity with sports scientist (PASS) programme in primary care: a cluster-randomised controlled trial. BMJ Open Sport Exerc Med. (2024) 10:e001985. doi: 10.1136/bmjsem-2024-001985 PMC11002402 38601124

[23] Silva DA Lima MR Tremblay MS Silva RJ. Academia da Saúde programme and physical activity levels in Brazilian adults: a nationwide cross-sectional study. J Phys Act Health. (2019) 16:772–9. doi: 10.1123/jpah.2018-0623, 31365900 PMC8883602

[24] Schneider M Gopfert A Iverson T Lemeunier N Woolf AD. National musculoskeletal policies: a comparative review of policy approaches, priorities, and implementation. Health Policy. (2023) 127:480–7. doi: 10.1016/j.healthpol.2023.03.001

[25] Tsuji I. Current status and issues concerning health Japan 21 (second term). Nutr Rev. (2020) 78:14–7. doi: 10.1093/nutrit/nuaa079, 33259610

[26] World Health Organization. World Report on Ageing and Health. Geneva: WHO (2015).

[27] Sundhedsstyrelsen. Physical Activity Guidelines for older Adults: Recommendations for Strength, Balance, and Mobility. Copenhagen: Danish Health Authority (2018).

[28] Nguyen HQ Ackermann RT Berke EM Cheadle A Lin E Williams B . Impact of a medicare physical activity benefit on health care utilization and costs in older adults. Prev Chronic Dis. (2008) 5:A0918081998

[29] Prado CM Laviano A Gill T Sanchez AM Jager-Wittenaar H Stephan BCM . Advances in muscle health and nutrition: a toolkit for clinical and public health practice. Clin Nutr. (2022) 41:2564–78. doi: 10.1016/j.clnu.2022.06.012

[30] Álvarez-Bustos A Rodríguez-Sánchez B Carnicero-Carreño JA Sepúlveda-Loyola W García-García FJ Rodríguez-Mañas L. Healthcare cost expenditures associated to frailty and sarcopenia. BMC Geriatr. (2022) 22:747. doi: 10.1186/s12877-022-03439-z 36096728 PMC9469617

[31] Janssen I Shepard DS Katzmarzyk PT Roubenoff R. The healthcare costs of sarcopenia in the United States. J Am Geriatr Soc. (2004) 52:80–5. doi: 10.1111/j.1532-5415.2004.52014.x, 14687319

[32] Bruyère O Beaudart C Ethgen O Reginster JY Locquet M. The health economics burden of sarcopenia: a systematic review. Maturitas. (2019) 119:61–9. doi: 10.1016/j.maturitas.2018.11.003, 30502752

[33] Burton E Farrier K Lewin G Pettigrew S Hill AM Airey P . Motivators and barriers for older people participating in resistance training: a systematic review. J Aging Phys Act. (2017) 25:311–24. doi: 10.1123/japa.2015-0289, 27620535

[34] World Health Organization. Decade of Healthy Ageing 2021–2030. Geneva: WHO (2020).

[35] World Health Organization. Global status Report on Physical Activity 2022. Geneva: WHO (2022).

[36] Groessl EJ Kaplan RM Rejeski WJ Katula JA Glynn NW King AC . Cost-effectiveness of the LIFE Physical Activity Intervention for Older Adults at Increased Risk for Mobility Disability. J Gerontol A Biol Sci Med Sci. (2016) 71:656–62. doi: 10.1093/gerona/glw029 26888433 PMC5007742

